# Comparison of PCR methods for detection of *Leishmania siamensis* infection

**DOI:** 10.1186/s13071-014-0458-x

**Published:** 2014-10-02

**Authors:** Atitaya Hitakarun, Peerapan Tan-ariya, Suradej Siripattanapipong, Mathirut Mungthin, Phunlerd Piyaraj, Tawee Naaglor, Padet Siriyasatien, Saruda Tiwananthagorn, Saovanee Leelayoova

**Affiliations:** Department of Microbiology, Faculty of Science, Mahidol University, Ratchawithi Rd., Ratchathewi, Bangkok 10400 Thailand; Department of Parasitology, Phramongkutklao College of Medicine, Bangkok, 10400 Thailand; Department of Parasitology, Faculty of Medicine, Chulalongkorn University, Bangkok, 10330 Thailand; Department of Veterinary Biosciences and Veterinary Public Health, Faculty of Veterinary Medicine, Chiang Mai University, Chiang Mai, 50100 Thailand

**Keywords:** *Leishmania siamensis*, Polymerase chain reaction, Sensitivity, Specificity, The internal transcribed spacer 1 region

## Abstract

**Background:**

*Leishmania siamensis*, a newly identified species, has been reported as a causative agent of leishmaniasis in Thailand. This organism has been identified and genetically characterized using PCR techniques based on several target genes. However, the sensitivities and specificities of these methods for the diagnosis of *L. siamensis* infection have never been evaluated.

**Methods:**

To evaluate the sensitivities and specificities of PCR methods to detect *L. siamensis* infection, PCR for different genetic markers, *i.e*., the small subunit ribosomal RNA region (SSU-rRNA), the internal transcribed spacer 1 region (ITS1), *cysteine protease* B (*cpb*), *cytochrome* b (*cyt* b), heat shock protein 70 (*hsp*70), the spliced leader mini-exon, and the triose-phosphate isomerase (*tim*) genes were compared*.*

**Results:**

Both the ITS1-PCR and the SSU rRNA-PCR could detect promastigote of *L. siamensis* at concentrations as low as 0.05 parasites/μl or the DNA concentration at 2.3 pg/μl. Though the ITS1-PCR method only recognized 8 samples as positive, all of these could be assessed as true positive according to microscopic diagnosis and/or amplifying the results of the PCR and their sequencing, while other methods also produced false positive results. Compared with the ITS1-PCR method, the PCR amplified SSU-rRNA and *cpb* gene showed 100% sensitivity for the detection of *L. siamensis* in clinical specimens. The PCR amplified mini-exon and *hsp*70 gene also gave a high sensitivity of 87.5%. In contrast, the PCR methods for *cyt* b and *tim* gene showed low sensitivity. The PCR methods for *cyt* b, mini-exon and *tim* gene showed 100% specificity compared with the ITS1-PCR.

**Conclusion:**

As a result, the ITS1-PCR method is a suitable target for PCR-based detection of *L. siamensis* infection in clinical specimens due to its high sensitivity and specificity. The results of this study can be used for molecular diagnosis as well as in epidemiological studies of *L. siamensis* in affected areas.

## Background

Leishmaniasis, one of the most important vector-borne diseases, has been reported worldwide with an estimated 500,000 new cases and more than 50,000 deaths per year [1]. *Leishmania siamensis*, a species recently reported in Thailand, causes both cutaneous and visceral leishmaniasis especially in patients with immunocompromised conditions such as HIV/AIDS [2–6]. In addition, leishmaniasis caused by *L. siamensis* was also reported in animals [7–9]. Conventional diagnostic methods based on microscopic examination as well as parasite cultures from clinical specimens such as buffy coat, bone marrow and spleen aspirates show a limited sensitivity [10,11]. Serological diagnostic methods also give a high sensitivity; however cross-reactivity could occur with other kinetoplastid parasites. These methods also fail to distinguish between past and present infection [12]. Accordingly, PCR methods have been widely used to detect *Leishmania* infection in clinical specimens with a high sensitivity and specificity [13]. Several PCR approaches have been published based on different target genes, *i.e*., the *cysteine protease* B (*cpb*) [14], the *cytochrome* b (*cyt* b) [15], the internal transcribed spacer 1 (ITS1) region of the small subunit ribosomal RNA (SSU-rRNA) gene [16], the glucose-6-phosphate dehydrogenase (G6PD) [17], the *heat shock protein* 70 (*hsp*70) [18], the spliced leader mini-exon [19], the SSU-rRNA gene [20] and the *triose-phosphate isomerase* (*tim*) genes [21]. Of these, the ITS1 region of the SSU-rRNA gene has been one of the common genetic markers used to detect *Leishmania* species in the Old World [13,16,22–24].

*L. siamensis* have been identified and genetically characterized using several target genes [2–6]. However, diagnosis of *L. siamensis* infection using PCR methods based on different genetic markers has never been evaluated. Thus, this study aimed to compare the sensitivity and specificity of different PCR methods, *i.e*., the *cpb*, the *cyt* b, the *hsp*70, the spliced leader mini-exon, the SSU-rRNA, the *tim* and the ITS1-PCR method.

## Methods

### *Leishmania* parasite

Promastigotes of *L. siamensis* (MHOM/TH/2010/TR), harvested from axenic culture in Schneider’s *Drosophila* medium, supplemented with 20% fetal bovine serum (FBS), were used in this study [25]. Promastigotes were centrifuged at 2,000 *g* for 15 min, washed three times with phosphate buffered saline (PBS), and resuspended in 1 ml of PBS. Numbers of parasites were counted and calculated using a hemocytometer. DNA was extracted using Illustra tissue & cells genomic Prep Mini Spin Kit (GE Healthcare, UK). DNA extraction from promastigotes of *L. donovani* strain MHOM/SD/68/1S (Biomedical Research, Amsterdam, The Netherlands) was also performed and used as a reference strain in this study. All extracted DNA samples were stored at −20°C until used.

### PCR assay

Table 1 shows different primer pairs used to detect *L. siamensis* in this study, which amplified different target genes, *i.e*., the *cpb*, the *cyt b*, the ITS1 region of the SSU-rRNA, the *hsp*70, the spliced leader mini-exon, the SSU-rRNA and the *tim* gene. To amplify the *cyt b* [15], the ITS1 region of SSU-rRNA [16], the mini-exon [19] and the SSU-rRNA gene [20], the previously described primer pairs were used. For the *hsp*70 gene, the HSP70senKS primer was designed and replaced the HSP70sen of the reported primer set (HSP70sen/HSP70ant) [18] to increase the sensitivity to detect *L. siamensis*. In addition, new primer pairs for the *cpb* (LH-CPBEF-F2/LH-CPBEF-R) and *tim* (TIM-F/TIM-R) gene were designed and used in this study.

**Table 1:** **PCR methods and primers used in this study**

**Target gene**	**Primer set**	**Primer sequence 5′-3′**	**Annealing (°C)**	**Expected amplicon size (bp)**	**Ref.**
**ITS1**	L5.8S	TGA TAC CAC TTA TCG CAC TT	53	348	[13]
	LITSR	CTG GAT CAT TTT CCG ATG			
***cpb***	LH-CPBEF-F2	TGC GGS TCS TGC TGG GCS TTC	59	525	This study
	LH-CPBEF-R	GCG SAY GTA SCC CTT CTC RC			
***cyt b***	L.cyt-S	GGT GTA GGT TTT AGT YTA GG	55	900	[12]
	L.cyt-R	CTA CAA TAA ACA AAT CAT AAT ATR CAA TT			
***hsp70***	HSP70senKS	GAC GGT GCC KGC STA CTT CAA	61	1422	[15], This study
	HSP70ant	CCG CCC ATG CTC TGG TAC ATC			
**mini-exon**	Fme	TAT TGG TAT GCG AAA CTT CCG	54	540	[16]
	Rme	ACA GAA ACT GAT ACT TAT ATA GCG			
**SSU-rRNA**	S4	GAT CCA GCT GCA GGT TCA CC	67	540	[17]
	S12	GGT TGA TTC CGT CAA CGG AC			
***tim***	TIM-F	TCA ACG AKC ATC AGA TCG AC	53	500	This study
	TIM-R	ATC TTC TCG CTC ACC CAC TG			

All PCR methods were performed at a final volume of 50 μl, containing 25 pmol of each primer, 0.2 mM dNTP, 2 mM MgCl_2_, 1x PCR buffer, 1.5 U of *Taq* DNA polymerase and 1 μl of DNA template, using the optimal annealing temperature (Table 1). Extracted DNA from normal human blood was used as the internal control and distilled water was used as the negative control. Expected amplicons of PCR products were visualized using electrophoresis at 100 V on 1.5% agarose gel mixed with SYBR® Safe DNA gel stain (Invitrogen, USA) in 1X Tris Borate EDTA (TBE) buffer.

### Detection limit of PCR methods

Detection limit of all PCR methods was performed to detect *L. siamensis*. Ten-fold serial dilutions were performed to obtain samples containing 500, 50, 5, 0.5, 0.05, and 0.005 parasites/μl. DNA was then extracted using Illustra tissue & cells genomic Prep Mini Spin Kit (GE Healthcare, UK). Further PCR steps were performed using 1 μl of DNA template, containing the equivalent of the above dilutions. Another set of DNA templates was also used. DNA concentration was measured using a Nano Drop spectrophotometer (ND-1000 model, Thermo Scientific, USA). Ten-fold serial dilutions of the extracted DNA were then performed to obtain the DNA concentration at 23,000, 2,300, 230, 23, 2.3, and 0.23 pg/μl. One microliter of each concentration was used as DNA template for PCR amplification. The most sensitive method was defined as the method that could amplify the DNA extracted from the lowest number of promastigotes or the lowest DNA concentration of *L. siamensis*. In addition, DNA samples of *Trypanosoma brucei*, *T. cruzi*, *T. evansi*, *Trichomonas vaginalis*, *Giardia intestinalis*, *Plasmodium falciparum*, and *Entamoeba histolytica* were also test for the specificity of each PCR method.

### Sensitivities and specificities of PCR methods to detect *L. siamensis*

All 7 PCR methods were then evaluated for their sensitivities and specificities to detect *L. siamensis* in 42 clinical specimens. These clinical samples were sent to the Department of Parasitology, Phramongkutklao College of Medicine for microscopic and PCR diagnosis of leishmaniasis. Most samples were collected from the patients who presented with fever, hepatosplenomegaly and pancytopenia at tertiary hospitals in the South, the endemic areas of leishmaniasis in Thailand. Extracted DNA of 42 clinical samples from patients including buffy coat, bone marrow and tissue biopsy (one skin biopsy) were used. Of these, eight samples were assessed as true positive according to the results of the PCR amplifying the ITS1 region of the SSU-rRNA gene and their sequencing. Thirty four samples were considered as true negative because ITS1-PCR showed negative result. Of eight positive samples, three samples from bone marrow aspiration and one sample of skin biopsy were microscopically positive for amastigotes and ITS1-PCR positive for *Leishmania*. The other four positive samples were diagnosed by ITS1-PCR amplification using buffy coat samples. All eight samples were confirmed as *L. siamensis* infection by DNA sequencing of the ITS1 region of the SSU-rRNA gene. Sensitivities and specificities of six PCR methods to detect *L. siamensis* were determined comparing to the ITS1-PCR method.

### DNA sequencing

All positive PCR products from any PCR assays were confirmed for *L. siamensis* sequence identity. Bidirectional DNA sequencing was performed by the 1st Base Pte., Ltd., Singapore. Nucleotide sequences were validated using the Bioedit Program version 7.0.1.

### Statistical analysis

Sensitivities, specificities and positive and negative predictive values were calculated using two-by-two tables and StataCorp. 2009, Stata Statistical Software: Release 11. College Station, TX: StataCorp LP.

### Ethics statement

This study was approved by the Ethics Committee of the Royal Thai Army Medical Department, Thailand. No information on the patients was presented in this study.

## Results

### Detection limits of PCR methods

Table 2 shows the detection limit of the seven PCR methods. In this study, the ITS1- and the SSU-rRNA-PCR were the most sensitive methods that could detect *L. siamensis* at the concentration as low as 2.3 pg/μl or 0.05 parasites/μl. In contrast, five other PCR methods could amplify when higher concentrations of *L. siamensis* DNA were applied. The PCR method amplifying the ITS1 region of *L. siamensis* showed the amplicon of 348 bp, while amplifying *L. donovani* DNA generated the amplicon of 319 bp.

**Table 2:** **The detection limit of seven PCR methods for the detection of** ***L. siamensis***

**Target gene**	**PCR results by**											
	Number of parasites/μl*						**DNA concentration (pg/μl)**					
	**500**	**50**	**5**	**0.5**	**0.05**	**0.005**	**23000**	**2300**	**230**	**23**	**2.3**	**0.23**
**ITS1**	+	+	+	+	+	-	+	+	+	+	+	-
***cpb***	+	+	+	-	-	-	+	+	+	-	-	-
***cyt b***	+	+	+	-	-	-	+	+	+	-	-	-
***hsp70***	+	+	+	+	-	-	+	+	+	+	-	-
**mini-exon**	+	+	+	+	-	-	+	+	+	+	-	-
**SSU-rRNA**	+	+	+	+	+	-	+	+	+	+	+	-
***tim***	+	+	+	+	-	-	+	+	+	+	-	-

*Ten-fold serial dilutions were performed to obtain samples containing 500, 50, 5, 0.5, 0.05 and 0.005 promastigotes/μl before DNA extraction.

### Cross-amplification of PCR methods using DNA of other parasites

We determined the cross-amplification of these seven PCR methods using the DNA of other parasites. PCR amplifications of the mini-exon and the *tim* genes amplified no other parasites’ DNA (Table 3). The PCR method for the *cyt b* gene showed cross-amplification with *T. brucei* DNA. Both PCR methods targeting the SSU-rRNA and *hsp*70 genes could amplify the DNA of *T. brucei*, *and T. evansi*. The PCR method for the *cpb* gene amplified all the DNA of flagellates used in this study. The L5.8S/LITSR primers amplifying the ITS1 region could not amplify DNA of other parasites at 348 bp. A band at 600 bp appeared when amplifying *T. evansi* DNA. However, in contrast to the cross reactions produced by the other PCR methods, the positive band of *T. evansi* could easily be differentiated from that of *L. siamensis* by their distinctive sizes.

**Table 3:** **Comparison of cross-amplification of seven PCR methods**

**Target gene**	**PCR results**						
	***T. brucei***	***T. cruzi***	***T. evansi***	***T. vaginalis***	***G. intestinalis***	***P. falciparum***	***E. histolytica***
**ITS1**	-	-	+	-	-	-	-
***cpb***	+	+	+	+	+	-	-
***cyt b***	+	-	-	-	-	-	-
***hsp70***	+	-	+	-	-	-	-
**mini-exon**	-	-	-	-	-	-	-
**SSU-rRNA**	+	-	+	-	-	-	-
***tim***	-	-	-	-	-	-	-

### Sensitivities and specificities of PCR methods to detect *L. siamensis* in clinical samples

Table 4 shows the sensitivities, specificities and positive (PPVs) and negative predictive values (NPVs) of six PCR methods compared with the ITS1-PCR to detect *L. siamensis* in clinical samples. PCR amplification of the ITS1 region rated 8 samples as positive, amplification of the *cpb* gene rated 24 samples as positive, both amplification of the SSU-rRNA and the *hsp*70 gene rated 14 samples as positive. DNA sequencing was performed for all positive PCR amplification. False positive was considered when the results of DNA sequencing were not compatible to *L. siamensis*. From these results, PCR amplification of the SSU-rRNA and the *cpb* gene showed the sensitivity of 100%. Both PCR method for the mini-exon and the *hsp*70 genes showed the sensitivity of 87.5%. PCR method for the *cyt b* gene showed the lowest sensitivity for the detection of *L. siamensis*. The specificity of 100% was also observed for PCR amplified the *cyt b*, the mini-exon and the *tim* gene.

**Table 4:** **Sensitivities, specificities and positive and negative predictive values of PCR methods to detect** ***L. siamensis*** **compared with the ITS1-PCR methods**

**Method**	**Result**	***L. siamensis***		**Sensitivity %**	**Specificity %**	**PPV %**	**NPV %**
		**+ve**	**-ve**				
		**no. (%)**	**no. (%)**	**(95% CI)**	**(95% CI)**	**(95% CI)**	**(95% CI)**
***cpb***	+ve	8 (100.0)	16 (47.1)	100 (63.1-100.0)	52.9 (35.1-70.2)	33.3 (15.6-55.3)	100 (81.5-100.0)
	-ve	0 (0)	18 (52.9)				
***cyt b***	+ve	3 (37.5)	0 (0)	37.5 (8.52-75.5)	100 (89.7-100.0)	100 (29.2-100.0)	87.2 (72.6-95.7)
	-ve	5 (62.5)	34 (100.0)				
***hsp70***	+ve	7 (87.5)	7 (22.8)	87.5 (47.3-99.7)	79.4 (62.1-91.3)	50 (23.0-77.0)	96.4 (81.7-99.9)
	-ve	1 (12.5)	27 (77.2)				
***mini-exon***	+ve	7 (87.5)	0 (0)	87.5 (47.3-99.7)	100 (89.7-100.0)	100 (59.0-100.0)	97.1 (85.1-99.9)
	-ve	1 (12.5)	34 (100.0)				
***SSU-rRNA***	+ve	8 (100.0)	6 (17.6)	100 (63.1-100.0)	82.4 (65.5-93.2)	57.1 (28.9-82.3)	100 (87.7-100.0)
	-ve	0 (0)	28 (82.4)				
***tim***	+ve	4 (50)	0 (0)	50 (15.7-84.3)	100 (90.7-100.0)	100 (39.8-100.0)	90.5 (77.4-97.3)
	-ve	4 (50)	38 (100.0)				

+ve, positive, −ve, negative.

## Discussion

The present study showed that the PCR amplifying the ITS1 region of SSU-rRNA gene (L5.8S/LITSR) was the most accurate method to detect *L. siamensis*. Though only 8 samples were recognized as positive, all of these could be assessed as true positive according to microscopic diagnosis and/or amplifying the results of the PCR and their sequencing, while other methods also produced false positive results. To date, there is still no gold standard technique for the diagnosis of leishmaniasis. Thus, the sensitivities and the specificities of other six PCR methods were determined by comparing to the ITS1-PCR method. Our result was similar to a recent study that tested the ITS1-PCR method in *L. donovani* [26]. This method could detect the promatigote of *L. siamensis* as low as 0.05 parasites/μl or a DNA concentration at 2.3 pg/μl. For clinical samples, 100% of *L. siamensis*-positive samples (eight samples) were identified by this method. Using the ITS1 region of the SSU-rRNA gene as the target for PCR showed a high sensitivity because approximately 20–400 copies of the SSU-rRNA gene per *Leishmania* genome exist [27–29]. The ITS1-PCR method can also differentiate *L. siamensis* from *L. donovani* in a single-tube PCR. The DNA of *L. siamensis* was detected at 348 bp whereas that of *L. donovani* was shown at 319 bp. Additionally, this primer pair could not amplify the DNA of other protozoa parasites, except *T. evansi*, which was detected at 600 bp. A study in Brazil also showed a cross-amplification of the ITS1-PCR method in *T. cruzi* showing the amplicon at 700 bp [30]. However, *L. siamensis* and *T. evansi* can be clearly discriminated by the size of their amplicons. The ITS1-PCR method also gave a negative result when human DNA was used as the internal control (data not shown).

The detection limit of S4/S12, the primer pair targeting the SSU-rRNA gene was compatible with the ITS1-PCR method. Although the SSU-rRNA PCR showed 100% sensitivity to detect *L. siamensis* in clinical samples, false positive results could be detected in six samples. DNA sequencing of these PCR products indicated that this primer pair could amplify human DNA. In addition, the S4/S12 primers could also amplify the DNA of other flagellate protozoa, *i.e.*, *T. brucei* and *T. evansi* and gave the amplicon at 563 bp. Thus, the SSU-rRNA PCR method to diagnose *Leishmania* infection in clinical specimens should be used with caution and the positive PCR products have to be confirmed by sequence analysis. Another disadvantage of the SSU-rRNA PCR method is that it cannot differentiate among the different species of *Leishmania* [13,25,30,31].

The Fme/Rme primer set, amplifying the mini-exon gene [19], could detect *L. donovani* at the DNA concentration as low as 10 pg/μl. The limit of this primer set to detect *L. siamensis* was in the same range as previously reported in *L. donovani* [32]. The mini-exon gene contains 100–200 copies in the nuclear *Leishmania* genome, consisting of transcribed conserved and non-transcribed variable regions [32]. These regions differ among *Leishmania* species. Thus, it could be used to differentiate *Leishmania* species. For clinical specimens, this primer set showed the sensitivity of 87.5% for the detection of *L. siamensis*. Although a previous study showed a cross-species amplification in *T. cruzi*, *T. brucei*, and *Crithidia fasciculata* [33], no amplification in a wide range of parasites and also human DNA was found in this study.

Montalvo et al. [34] reported a primer set specific to the *hsp*70 to detect *Leishmania*. This primer set showed different detection limits in different *Leishmania* subgenera. In the present study, the HSP70sen/HSP70ant primer set could amplify the DNA of *L. siamensis* at the lowest concentration of 230 pg/μl. Therefore, the HSP70senKS primer was designed and replaced the HSP70sen to increase the sensitivity showing the detection limit at 23 pg/μl. However, this newly designed as well as the previously described primer pairs [34] could detect other hemoflagellates. False positive result might also limit the use of HSP70 PCR. DNA sequencing of 7 false positive PCR products showed that the HSP70 PCR could amplify human DNA as well.

The cpbEF-frd/cpbEF-rev primer set has been used to amplify the *cpb* gene of *Leishmania* species, both *L. donovani* and *L. infantum* [35,36]. However, this primer set could not amplify the DNA of *L. siamensis*. Therefore, a new primer set (LH-CPBEF-F2/LH-CPBEF-R) was designed based on the *cpb* sequence data of all *Leishmania* species reported in GenBank. This new primer set amplified *L. siamensis* DNA and showed 100% sensitivity, equivalent to the ITS1 and SSU-rRNA PCR methods. Unfortunately, LH-CPBEF-F2/LH-CPBEF-R primer set could amplify the DNA of other flagellate parasites including *T. brucei*, *T. cruzi*, *T. evansi*, *T. vaginalis* and *G. intestinalis*. Among all tested PCR methods, the LH-CPBEF-F2/LH-CPBEF-R primer set gave the lowest specificity to detect *L. siamensis* in clinical specimens.

To date, the information on *tim* gene are limited because only a small number of sequences of *Leishmania* species were reported in GenBank. In the present study, a new set of primers amplifying the *tim* gene has been designed which could amplify *Leishmania* at the concentration as low as 0.5 parasites/μl or a DNA concentration at 23 pg/μl. However, the sensitivity of *tim*-PCR method was still lower compared with the ITS1-PCR method. The L.cyt-S/L.cyt-R primer set for the PCR method amplifying the *cytb* gene also showed a low sensitivity at 37.5%. Thus, these two PCR methods might not be suitable for the screening of *L. siamensis* infection.

The limitation of this study was that the number of patients/clinical samples used in this study was rather small and the 95%-CIs were therefore extremely wide. Increased accuracy of these tests may need a larger sample size. However, the acquisition of sufficient positive samples can be a troublesome labor and maybe the acquisition of higher number samples of this newly recognized parasite species was not possible.

## Conclusion

Among these tested PCR methods, the ITS1-based method is the most sensitive and suitable to diagnose leishmaniasis caused by *L. siamensis*. This PCR method could differentiate *L. siamensis* and *L. donovani* by their amplicon sizes in a single-tube PCR. Due to its high sensitivity, this method would be useful for the epidemiological survey of *L. siamensis* infection in Thailand.
